# Future directions in haploidentical hematopoietic stem cell transplantation

**DOI:** 10.1080/16078454.2024.2366718

**Published:** 2024-06-18

**Authors:** Pongthep Vittayawacharin, Piyanuch Kongtim, Stefan O. Ciurea

**Affiliations:** a Hematopoietic Stem Cell Transplantation and Cellular Therapy Program, Division of Hematology/Oncology, Department of Medicine, University of California, Irvine, CA, USA; b Division of Hematology, Department of Medicine, Faculty of Medicine Siriraj Hospital, Mahidol University, Thailand

**Keywords:** Haploidentical hematopoietic stem cell transplantation, younger haploidentical donor, NK cell alloreactivity, KIR, buffy coat, donor-specific anti-HLA antibodies, cellular therapy, chiemeric antigen recepter T-cells

## Abstract

Outcomes of haploidentical hematopoietic stem cell transplantation (haplo-SCT) have improved over time. Graft failure and graft-versus-host disease (GVHD), which were important complications in major human leukocyte antigen (HLA)-disparity stem cell transplantation, have significantly decreased. These improvements have led to an exponential increase in the use of haploidentical donors for transplantation, as well as in the number of publications evaluating haplo-SCT outcomes. Many studies focused on factors important in donor selection, novel conditioning regimens or GVHD prophylaxis, the impact of donor-specific anti-HLA antibodies (DSA), as well as strategies to prevent disease relapse post-transplant. DSA represents an important limitation and multimodality desensitization protocols, including plasma exchange, rituximab, intravenous immunoglobulin and donor buffy coat infusion, can contribute to the successful engraftment in patients with high DSA levels and is currently the standard therapy for highly allosensitized individuals. With regards to donor selection, younger donors are preferred due to lower risk of complications and better transplant outcomes. Moreover, recent studies also showed that younger haploidentical donors may be a better choice than older-matched unrelated donors. Improvement of disease relapse remains a top priority, and several studies have demonstrated that higher natural killer (NK) cell numbers early post-transplant are associated with improved outcomes. Prospective studies have started to assess the role of NK cell administration in decreasing post-transplant relapse. These studies suggest that the incorporation of other cell products post-transplant, including the administration of chimeric antigen receptor T-cells, should be explored in the future.

## Introduction

Haploidentical hematopoietic stem cell transplantation (haplo-SCT) has been increasingly performed as the treatment of high-risk hematologic diseases, due to lower cost and rapid availability of a haploidentical donor [1]. Selecting the appropriate donor for transplantation becomes more important when more than one such donor is available. Significant human leukocyte antigen (HLA) disparity is a major barrier, which can result in higher treatment-related mortality (TRM), due to graft failure and graft-versus-host disease (GVHD). However, the use of post-transplant cyclophosphamide (PTCy) has successfully mitigated these complications, is now widely applied in this type of transplant, and its use extends to transplantation with other donor types [2]. Donor-specific anti-HLA antibodies (DSA) represent a major limitation in transplantation with HLA mismatched donors and are more relevant in haplo-SCT due to higher prevalence in allosensitized multiparous female recipients and transplantation with a child as a donor that shares other parent’s HLA antigens. Detection of significant levels of DSA in such recipients requires desensitization therapy in order to ensure successful engraftment and prevent TRM [3,4]. Despite significant improvements in haplo-SCT procedures, disease relapse remains a significant cause of treatment failure [5]. Several strategies have been evaluated for prevention of disease relapse, including maintenance with targeted drug therapy, or adoptive cellular therapy.

This review focuses on the current evidence and explores future directions in haplo-SCT, with regards to optimizing engraftment by mitigating DSAs, donor selection considering donor age and donor-recipient natural killer (NK) cell alloreactivity, as well as prevention of disease relapse after haplo-SCT using cellular therapy, which is arguably the most important factor that could significantly impact transplant outcomes and further promote transplantation as a curative procedure for patients with hematological malignancies.

## Donor selection

Several potential haploidentical donors are commonly available for transplantation. This raises the question: apart from DSA, which donor factors have a significant impact on transplant outcomes? Such factors include ABO compatibility, cytomegalovirus serostatus, donor age, donor relationship, NK cell alloreactivity and killer Ig-like receptor (KIR) haplotype. Donor age remains one of the important factors considered by transplant physicians to improve transplant outcomes [6-8]. The optimal age for haploidentical donors is unclear. Younger donors are preferred due to lower TRM and lower risk of clonal somatic mutations in hematopoietic cells [9]. Furthermore, the role of donor-recipient NK cell alloreactivity remains controversial. Herein, we reviewed current evidence on the impact of donor age and donor-recipient KIR typing, which could have a significant impact on haplo-SCT outcomes.

### Donor age: younger haploidentical donor or older fully matched-HLA donor

DeZern and colleagues recently reported results from a retrospective analysis of 889 patients who underwent nonmyeloablative (NMA) haplo-SCT and demonstrated that an increase in donor age by 10 years was associated with inferior progression-free survival (PFS) and inferior overall survival (OS), due to higher non-relapse mortality (NRM), related to an elevated risk of grade 2–4 acute GVHD (aGVHD). Relapse rate and incidence of chronic GVHD (cGVHD) were not affected by increasing donor age [6]. The optimal age appears to be less than 30–40 years old. This could be more relevant for older patients who are less likely to tolerate aGVHD. Ciurea et al. also reported that donor age <40 years in patients with acute myeloid leukemia and myelodysplastic syndromes (AML/MDS) was associated with better survival [10]. Canaani et al. showed that donors <40 years in patients with AML/acute lymphoblastic leukemia (ALL) should be preferred due to improved OS [11]. In addition, Wang and colleagues reported that donors below the age of 30 correlated with reduced NRM and improved OS [12]. Recently, Saliba et al. reported on the impact of donor age in AML patients receiving a haplo-SCT with different intensity of conditioning regimens (*N* = 790). In the myeloablative conditioning regimen, donor age > 35 years was associated with a higher risk of cGVHD and higher overall mortality 6 months after transplantation in recipients age ≤ 50 years. In reduced-intensity or NMA conditioning, donor age > 35 years was associated with higher NRM (HR 1.6, *P* = 0.04) and high overall mortality after 9 months post-transplantation in recipient age > 50 years [7]. These results suggested that a donor age of approximately < 40 years is associated with improved survival.

The Center for International Blood and Marrow Transplant Research (CIBMTR) compared younger haploidentical donors with PTCy (≤35 years) and older matched-unrelated donors (MUD) with conventional GVHD prophylaxis (>35 years) in two cohorts of patients with AML/MDS and ALL, and both studies favored the younger haploidentical donors. In the AML/MDS cohort, 494 haplo-SCT patients and 1,005 MUD transplant patients were included. Younger haploidentical donors showed superior 4-year OS (HR 0.81, *P* = 0.01), lower cumulative incidence of 4-year NRM (HR 0.59, *P* = 0.02) due to lower incidence of 100-day grade 2–4 aGVHD (HR 0.64, *P* < 0.001), and lower incidence of 2-year cGVHD (HR 0.49, *P* < 0.001) [13]. In the second analysis of ALL patients with younger haploidentical donors (*N* = 187) and older MUD donors (*N* = 232), transplantation with older MUDs was associated with a higher incidence of 2-year cGVHD (HR 1.91, *P* = 0.002), higher incidence of 4-year NRM (HR 2.75, *P* = 0.001) and inferior 4-year OS (HR 1.77, *P* = 0.08) [14].

### Donor–recipient natural killer cell alloreactivity and KIR haplotype

NK cell alloreactivity, resulting from the interaction between inhibitory and activating receptors expressed on the NK cell surface, may enhance the graft-versus-leukemic effect in post-transplantation [15]. The ‘missing self’ hypothesis initially described the mechanism of NK cell-mediated cell lysis which occurs when HLA class I-specific inhibitory receptors on NK cells fail to detect HLA class I on the target cell [16]. Activation of NK cells can also occur from other activation receptors [17,18]. The killer Ig-like receptors (KIR) are inhibitory receptors that can recognize self from non-self by interacting with MHC class I molecules. KIRs exhibit either 2 or 3 extracellular immunoglobulin domains, called KIR2D and KIR3D, respectively. For intracytoplasmic structures, KIRs have either short (S) or long (L) intracytoplasmic tails that function as activating or inhibitory signals, respectively. The inhibitory KIRs, as discussed above, recognize HLA class I as a ligand and demonstrate specificity between the type of inhibitory KIRs and specific HLA class I [19]. If an NK cell expresses an inhibitory KIR that does not recognize a specific HLA class I on the target cell (KIR mismatch), the target cell is eliminated, as described by the receptor–ligand model [20]. The KIR genes are in the leukocyte receptor complex on human chromosome 19, and the KIR haplotypes are classified into two distinct groups: A and B haplotypes. The A haplotype has a smaller number of genes that encode only inhibitory receptors, except for KIR2DS4. In contrast, the B haplotype contains a greater number of genes that encode both inhibitory and activating receptors. All individuals can be classified into one of two KIR genotypes: either group A KIR haplotypes (A/A) or group B haplotype (B/x) [19]. Both donor-recipient NK cell alloreactivity and KIR haplotype have been evaluated as one of the factors associated with post-haploidentical transplant outcomes. There has been conflicting evidence regarding the impact of different NK alloreactive models on transplant outcomes [21]. Some studies have demonstrated positive outcomes from donor-recipient NK cell alloreactivity, inhibitory KIR gene mismatch, KIR receptor–ligand mismatch and KIR haplotype B donors [22-25], while others have shown no difference or have resulted in negative transplant outcomes [26-28].

Two recent studies have demonstrated that a higher Count Functional inhibitory KIR score (CF-iKIR), which represents the sum of functional KIRs with corresponding ligands on target cells (CF-iKIR = 1 for KIR2DL1, + 1 for functional KIR2DL2 and/or KIR2DL3, and +1 for functional KIR3DL1), is associated with improved outcomes in both matched unrelated donor and haploidentical donor transplants. Zou and colleagues conducted a retrospective study on 354 patients who received haplo-SCT with PTCy and showed that a CF-iKIR score >2 was associated with improved PFS and OS, while, all other NK cell alloreactivity models, including donor NK cell benefit (KIR-ligand mismatch), KIR2DS1/C1C2 epitope combination, donor centromeric motif, donor telomeric motif, KIR B-content score, and inhibitory KIR score, did not show correlations with survival outcomes [8]. In addition, this model proved to be associated with a lower risk of relapse and superior event-free survival in a cohort of 1,704 MDS or secondary AML patients receiving MUD transplantation [29]. These results aligned with a recent paper from the European Society for Blood and Marrow Transplantation (EMBT), which showed that KIR ligand mismatch was associated with higher NRM and worse survival in haplo-SCT [28]. Zou et al. found this model to be at the opposite end compared with CF-iKIR in the heatmap correlation of all KIR models [8,29]. Corroborating findings from all these 3 studies, KIR alloreactivity as assessed by CF-iKIR score, rather than KIR-ligand mismatch, is associated with better outcomes in haplo-SCT with PTCy. Further studies will be needed to confirm these findings.

## Treatment of patients with donor-specific anti-HLA antibodies

PTCy-based GVHD prophylaxis can overcome HLA-disparity in haplo-SCT, providing alternative donor options for almost all patients without an HLA-matched donor [30,31]. However, DSA remains a significant barrier to haplo-SCT. DSA has been associated with delayed neutrophil and platelet recovery, as well as primary graft failure, with a significantly negative impact on transplant outcomes [32,33]. Transplants with DSA levels above 20,000 mean fluorescence intensity (MFI) or persistent positive C1q at the time of transplantation are at the highest risk of engraftment failure, have poor survival and, so far, no reliable treatment exists for these patients [34]. In patients with DSA up to 20,000 MFIs, desensitization therapy using multimodality of procedures and agents, including plasma exchange (PEX) to eliminate antibodies, rituximab to inhibit antibody production, intravenous immunoglobulin (IVIG), as well as infusion of an irradiated donor-derived buffy coat prepared from donor’s mononuclear cells, or HLA class I compatible platelets for neutralizing antibodies could be effective in eliminating the DSA and achieve engraftment [3].

**Figure 1.**
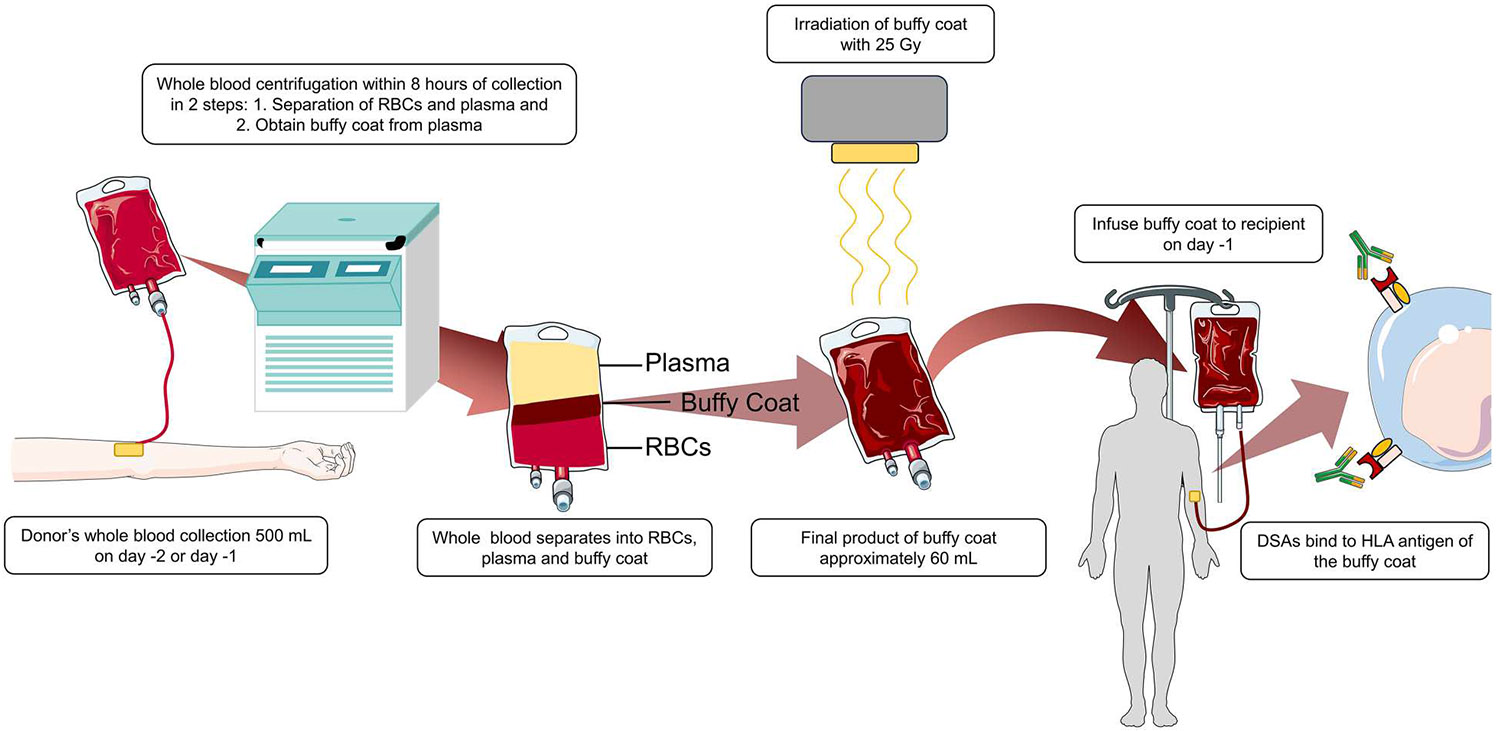
Buffy coat preparation.

Infusion of the irradiated buffy coat has been shown to have a significant role in improving engraftment [34]. The buffy coat is prepared using the centrifugation method from one unit of blood and contains granulocytes, lymphocytes, monocytes, which express HLA class I and class II molecules, while platelets express only HLA class I molecule [35,36]. The neutralization of DSA results from the binding of corresponding HLA antigens from donor-derived buffy coat and DSA present in the recipient’s serum, preventing binding to HLA antigens of stem cells, infused one day later (Figure 1).

Ciurea and colleagues recently reported outcomes of patients with DSA with desensitization protocol, including PEX with either fresh frozen plasma or albumin at 1–1.5 total plasma volume for three alternate day sessions, rituximab 375 mg/m^2^ single dose on the day after complete PEX, IVIG 1 g/kg on the day after rituximab and donor-derived irradiated buffy coat infused on day −1 in 37 patients with mean pre-desensitization DSA at 10,198 MFI. The buffy coat was administered to 20 patients (77.1%). Fourteen out of 30 evaluated patients for C1q (46.7%) had positive results. The mean post-desensitization DSA level was 5397 MFI and 8 out of 29 evaluated pre-transplant C1q remained positive (27.6%). The cumulative incidence of neutrophil engraftment at 28 days and platelet engraftment at 60 days were 75.7% and 58.9%, respectively. Compared with a control group of patients without DSA (*N* = 345), there was no significant difference between the two groups in terms of engraftment, PFS, OS, relapse and NRM. However, the subgroup of patients with DSA >20,000 MFI and C1q persistently positive after desensitization had a higher engraftment failure rate, higher TRM and worse survival compared with the control group. The cumulative incidence of neutrophil engraftment in the buffy coat group was superior to that in the group of desensitized patients without buffy coat, after adjusting for DSA levels (subdistribution HR 2.09, *P* = 0.049) [34].

The Madrid Group of Hematopoietic Transplant reported haplo-SCT outcomes in 19 desensitized DSA-positive patients. Desensitization protocols varied among patients, including PEX, rituximab, IVIG, incompatible platelets, immunosuppressive agents (mycophenolate mofetil, tacrolimus, steroids), and donor buffy coat. Among the 19 patients, 31% (6 out of 19) received a buffy coat as part of the desensitization protocol, with a median initial DSA of 9000 MFI. On the day of stem cell infusion, all six patients had DSA levels less than 5000 MFI, without DSA rebound, and none of them experienced graft failure [37].

These results suggest that desensitization of patients with DSA up to 20,000 MFI is feasible and is associated with outcomes similar to patients without DSA.

## Prevention of relapse after haploidentical hematopoietic stem cell transplantation

Advancements in supportive care, donor selection and conditioning regimens have significantly reduced TRM over time with relapse emerging as a major cause of treatment failure, especially for patients with high-risk or advanced disease [5]. Because targeted therapy is available for a very limited group of patients, cellular therapies have been evaluated in this setting. Donor lymphocyte infusion (DLI) has demonstrated some efficacy in enhancing graft-versus-tumor effect from donor T-cells. A retrospective study by the EBMT group included 173 patients who underwent haplo-SCT with PTCy, of whom 34.3% of the participants received DLI as a prophylactic approach. The superiority of disease-free survival (DFS) and OS were observed in prophylactic DLI, compared with pre-emptive and therapeutic DLI. However, GVHD is still a main adverse event of this approach [38]. Due to this unmet need, novel cellular therapies, including NK cell therapy and chimeric antigen receptor (CAR) T-cell therapy, have been studied in this clinical setting.

### Natural killer cell therapy

Administration of NK cells after transplant may improve graft-versus-tumor effect without increasing GVHD [22,39,40]. Higher NK cell numbers in the early post-transpalnt period have been associated with improved transplant outcomes [15]. NK cells have been evaluated to improve disease relapse and survival post-transplant and, because of limited numbers in the apheresis product, *ex vivo* expansion with cytokines, mesenchymal stroma or genetically modified artificial antigen-presenting cells have been investigated to achieve higher doses of NK cells for administration [41-43]. Most studies involving adoptive NK cell therapy after transplantation are early clinical studies, focusing primarily on high-risk patients with myeloid malignancies; however, a recent randomized study confirmed the potential benefit of this approach.

Earlier studies using donor-derived NK cell infusion after haplo-SCT in very high-risk patients with refractory disease at the time of transplant showed poor outcomes with a high incidence of relapse and poor survival [44-47] (Table 1). Most subsequent studies focused on administrating NK cells to augment the antitumor effect for patients in remission at transplant. Ciurea and colleagues reported on the safety of membrane-bound (mb) interleukin (IL)-21 *ex vivo* expanded donor-derived NK cells infused early after haplo-SCT on days −2, + 7, and +28 after melphalan-based conditioning regimen. Thirteen patients with high-risk myeloid malignancies were included, with 1-year DFS 85%. There were no occurrences of infusion reactions, dose-limiting toxicities, or graft failure. None of the patients developed severe grade 3–4 aGVHD or extensive cGVHD [48]. A subsequent phase II study (*N* = 25) reported long-term follow-up results in patients with myeloid malignancies receiving haplo-SCT, comparing with case-matched cohort from the CIBMTR database. The study group demonstrated a 2-year relapse rate of only 4%, significantly lower than the control group of 38% (*P* = 0.014) with better 2-year DFS and OS (DFS 66% vs. 44% and OS 70% vs. 58%), yet not statistically significant presumably due to low number of patients. DFS was significantly improved when patients with DSA were excluded from analysis (adjusted HR 2.64, *P* = 0.029). Recipients of NK cells demonstrated elevated production levels of interferon-gamma and tumor necrosis factor-alpha and higher NK cell doses were associated with higher NK cells numbers detected early post-transplantation, suggesting an improvement in the anti-leukemic effect [49,50].

These results were recently confirmed in a phase II randomized clinical trial which included patients diagnosed with high-risk AML and MDS undergoing haplo-SCT. Patients were randomized into two groups: one receiving donor-derived *ex-vivo* expanded NK cells activated with IL-15 and IL-21 administered at day + 13 and +20 post-transplantation (*N* = 49), and a control group without NK cell therapy (*N* = 36). The study group showed a significantly lower cumulative incidence of disease progression at 30 months compared with the control group (35% vs. 61%, *P* = 0.040) yet no significant difference in PFS and OS was seen between the two groups; however, it is unclear if the study was adequately powered to address these endpoints [51].

### Chimeric antigen receptor T-cell therapy

Data on pre-emptive CAR T-cell therapy for patients with B-cell lymphoid malignancies after haplo-SCT is still very limited. Kebriaei et al. reported phase I study of a non-viral process using a Sleeping Beauty (SB) transposon/transposase system to generate CD19-specific CAR T-cells as an adjuvant therapy in high-risk B-cell ALL and non-Hodgkin lymphoma patients. Eight of 19 patients (42%) received a haploidentical donor for transplantation. CAR T-cells were administered after a median of 64 days post-transplant. The 1-year PFS and OS in haplo-SCT subgroup were 75% and 100%, respectively. CAR T-cells could be identified in peripheral blood at an average of 51 days [52]. While there is still no solid data regarding using commercial FDA-approved CAR T-cell therapy after allogeneic transplantation, several small case series demonstrated the safety and efficacy of axicabtagene ciloleucel as a treatment after disease relapse after transplant in NHL patients [53-55] (Table 2).

## Conclusions

Haploidentical donors have been successfully utilized as alternative donors for stem cell transplantation. Multiple retrospective studies have confirmed that younger donors may be associated with improved survival due to lower incidence of GVHD and NRM. An increase in NK cell alloreactivity with a higher number of inhibitory KIRs with corresponding ligands in recipient cells has shown favorable transplant outcomes. Early data using CF-iKIR needs to be validated in larger registry studies. Typing of KIR genes might be needed for donor selection in the future.

Patients with DSA, especially multiparous female patients, are at a higher risk for engraftment failure and require desensitization. Incorporating donor leukocyte irradiated buffy coat with a multimodality desensitization protocol demonstrated effectiveness in significantly reducing DSA levels and converting C1q to negative, which are prerequisites for stem cell engraftment. Future studies will explore using a larger volume of buffy coat to desensitize patients with very high DSA levels.

Preventing disease relapse with cellular therapy after haplo-SCT is a very promising treatment strategy with low toxicity, particularly for patients with high-risk features, detectable disease or positive measurable residual disease before transplantation. The graft of patients with myeloid malignancies could potentially be augmented with NK cells, while for those with B-cell malignancies with CAR T-cells administered early post-transplant.

## Figures and Tables

**Table 1.** Summary of natural killer cell therapy after haploidentical stem cell transplantation studies.

Study	Diagnosis	*N*	NK cell	Treatment	Outcomes	Toxicities
Stern M, et al. [47]	AML, ALL, HL, Sarcoma	16	Donor-derived NK cells	Mostly refractory disease	5-year OS 25%	Grade 2–4 aGVHD 4/16
Choi I, et al. [44]	AML, ALL, MDS, NHL	41	Donor-derived IL-15, IL-21 NK cells	Mostly refractory disease	r/r AML: EFS and OS 31% and 35% r/r lymphoid malignancies: EFS and OS 0%	CI of grade 2–4 aGVHD 17%
Choi I, et al. [45]	AML, ALL	51	Donor-derived IL-15, IL-21 NK cells	Mostly refractory disease	3-year EFS and OS: 9% and 21%	aGVHD overlapping CRS 9/51
Jaiswal SR, et al. [46]	AML, PMF, CML, MPAL	10	CD56-enriched DLI	Mostly refractory disease	OS 50%	No grade 2–4 aGVHD
Ciurea S, et al. [48]	AML, MDS, CML	13	mbIL21 ex vivo–expanded donor-derived NK cells	Augmented therapy with transplantation	1 yr DFS & OS: 85% & 92%	No grade 3–4 aGVHD or cGVHD
Ciurea S, et al. [49]	AML, MDS, CML	25	mbIL21 ex vivo–expanded donor-derived NK cells	Augmented therapy with transplantation	2 yr RR & DFS: 4% & 66%	Grade 2 aGVHD 9/24, Grade 4 aGVHD 1/24
Lee KH, et al. [51]	AML, MDS	40	donor-derived IL-15, IL-21 NK cells	Mostly refractory disease	30-month PFS &OS: 33% & 35%	CI of grade 2–4 aGVHD 51%

Abbreviation: aGVHD acute graft-versus-host disease, ALL acute lymphoblastic leukemia, AML acute myeloid leukemia, cGVHD chronic graft-versus-host disease, CI cumulative incidence, CML chronic myeloid leukemia, CRS cytokine release syndrome, DFS disease-free survival, DLI donor lymphocyte infusion, EFS event-free survival, HL Hodgkin lymphoma, IL interleukin, mb membrane-bound, MDS myelodysplastic syndromes, MPAL mixed-phenotype acute leukemia, NK natural killer, NHL non-Hodgkin lymphoma, OS overall survival, PFS progression-free survival, PMF primary myelofibrosis, RR relapse rate, r/r relapse/refractory

**Table 2.** Summary of chimeric receptor antigen T-cell therapy after haploidentical stem cell transplantation studies.

Study	Diagnosis	*N*	Type of CAR T-cell	Disease status	Outcomes	Toxicities
Kebriaei P, et al. [52]	ALL, NHL	8	CD19-specific CAR T cell, SB system	Refractory at haplo-SCT (5), remission at haplo-SCT (3)	1-year PFS & OS: 75% & 100%	Skin acute GVHD (1)
Jain T, et al. [53]	NHL	1	Axicabtagene ciloleucel	Relapse after haplo-SCT	CR at 270 days post CAR T-cell infusion	None
Schubert ML, et al. [54]	NHL	1	Axicabtagene ciloleucel	Relapse after haplo-SCT	PR	CRS I, ICANS IV
Lutfi F, et al. [55]	NHL	4	Axicabtagene ciloleucel	Relapse after haplo-SCT	PD (3), no evidence of disease (1)	CRS (3), ICANS (1)

Abbreviation: ALL acute lymphoblastic leukemia, CAR chiemeric antigen receptor, CR complete response, CRS cytokine release syndrome, GVHD graft-versus-host disease, haplo haploidentical, ICANS immune effector cell-associated neurotoxicity syndrome, NHL non-Hodgkin lymphoma, OS overall survival, PD progression disease, PFS progression-free survival, PR partial response, SB sleeping beauty transposon, SCT stem cell transplantation,

## Data Availability

Data sharing is not applicable as no datasets were generated for this review article.
